# The growth of assisted reproductive treatment-conceived children from birth to 5 years: a national cohort study

**DOI:** 10.1186/s12916-018-1203-7

**Published:** 2018-11-28

**Authors:** Mark Hann, Stephen A. Roberts, Stephen W. D’Souza, Peter Clayton, Nick Macklon, Daniel R. Brison

**Affiliations:** 1 Centre for Biostatistics, Division of Population Health, Health Services Research and Primary Care, School of Health Sciences, Faculty of Biology, Medicine and Health, University of Manchester, Oxford Road, Manchester, M13 9PL UK; 2 Maternal and Fetal Health Research Centre, University of Manchester, Manchester Academic Health Sciences Centre, Oxford Road, Manchester, M13 9PL UK; 3 Child Health and Paediatric Endocrinology, Division of Developmental Biology and Medicine, School of Medicine, Faculty of Biology, Medicine and Health, University of Manchester, Manchester Academic Health Sciences Centre, Oxford Road, Manchester, M13 9PL UK; 4 Human Development and Health Academic Unit, Faculty of Medicine, University of Southampton, Southampton, SO16 6YD UK; 5 Department of Obstetrics and Gynaecology, Princess Anne Hospital, Coxford Road, Southampton, SO16 5YA UK; 6 Department of Reproductive Medicine, Saint Mary’s Hospital, Manchester University NHS Foundation Trust, Manchester Academic Health Sciences Centre, Oxford Road, Manchester, M13 9PL UK

**Keywords:** Assisted reproductive technology, Birth weight, Child growth, Data linkage

## Abstract

**Background:**

Birth weight and early child growth are important predictors of long-term cardiometabolic disease risk, in line with the Developmental Origins of Health and Disease hypothesis. As human assisted reproductive technologies (ARTs) occur during the sensitive periconceptional window of development, it has recently become a matter of urgency to investigate risk in ART-conceived children.

**Methods:**

We have conducted the first large-scale, national cohort study of early growth in ART children from birth to school age, linking the register of ART, held by the UK’s Human Fertilisation and Embryology Authority, to Scottish maternity and child health databases.

**Results:**

In this study of 5200 ART and 20,800 naturally conceived (NC) control children, linear regression analysis revealed the birthweight of babies born from fresh embryo transfer cycles is 93.7 g [95% CI (76.6, 110.6)g] less than NC controls, whereas babies born from frozen embryo transfer (FET) cycles are 57.5 g [95% CI (30.7, 86.5)g] heavier. Fresh ART babies grew faster from birth (by 7.2 g/week) but remained lighter (by 171 g), at 6–8 weeks, than NC babies and 133 g smaller than FET babies; FET and NC babies were similar. Length and occipital-frontal circumference followed the same pattern. By school entry (4–7 years), weight, length and BMI in boys and girls conceived by fresh ART and FET were similar to those in NC children.

**Conclusions:**

ART babies born from fresh embryo transfer grow more slowly in utero and in the first few weeks of life, but then show postnatal catch up growth by school age, compared to NC and FET babies. As low birth weight and postnatal catch-up are independent risk factors for cardiometabolic disease over the life-course, we suggest that further studies in this area are now warranted.

**Electronic supplementary material:**

The online version of this article (10.1186/s12916-018-1203-7) contains supplementary material, which is available to authorized users.

## Background

More than six million children have been born worldwide since 1978 using assisted reproductive technologies (ART) [1]. While the majority of these appear to be healthy, a higher incidence of some congenital abnormalities, pre-term birth and low birth weight (LBW) for gestational age has been noted among ART singletons [2–5]. The latter is of increasing concern, since birth weight is a surrogate for fetal growth and a strong predictor of cardiometabolic disease (CMD) risk across the life-course [6] (the Developmental Origins of Health and Disease (DOHaD); https://dohadsoc.org/)).

Studies in both human populations [7] and animal models [8, 9] have clearly identified the periconceptional period immediately before and after fertilisation as a unique developmental window during which the long-term health of the individual may be programmed. This has raised significant concerns about ART children as it coincides with the period during which gamete and embryo manipulations take place in the non-physiological ART environment, with specific embryo manipulations associated with altered fetal growth and birth weight [9].

Postnatal infant and child growth is arguably an even more important indicator of long-term disease risk; in particular, catch-up growth from LBW increases the risk of obesity and CMD [10, 11]. Owing to the lack of routinely collected data, only a limited number of small (≤ 500 children) studies have been carried out on ART child growth and these generally show conflicting results. In three small European cohort studies, children born from intra-cytoplasmic sperm injection (ICSI) were found to be lighter than their target weight (for age and height) at age 3 years, but not at age 5 years [12]. A longitudinal study [13] noted that ART children had lower weight at birth and 3 months, with similar growth rates to age 3, compared with naturally conceived (NC) subfertile controls. Given the implications of altered early life growth for health risks later in life, it is clearly important to collect definitive data from larger groups of ART children. It is also far from clear which specific ART factors might be causing changes in birth weight, although extending the length of time embryos are kept in culture before (blastocyst) transfer, the type of culture medium and the use of frozen embryo transfer (FET) have been implicated [5, 14–17]. Without this essential information on modifiable risk factors, it will be impossible to improve the ART process and reduce long-term risk to children.

The UK pioneered ART 40 years ago with the first successful birth in Oldham in July 1978 and thus is home to the first cohort of children now approaching middle age. However, the UK has been among the slowest countries to conduct long-term ART follow-up studies, because the original Human Fertilisation and Embryology Act (1990) did not envisage this. In line with shifts in public opinion, the Act was changed in 2010 and under the principle of presumed consent, the UK now possesses the world’s oldest and largest database of ART available for research, with approximately 110,000 children from 1991 to 2009. However, so far, this has only been exploited for a single cancer follow-up study [18]. There have been no large studies of ART child growth or causal factors in the UK or anywhere in the world. Therefore, the primary aim of this work was to assess the role of ART treatment factors on growth from birth to age of school entry, in a large UK national study. A secondary aim was to provide comparative data for NC babies.

## Methods

### Data sources

**Fig. 1:**
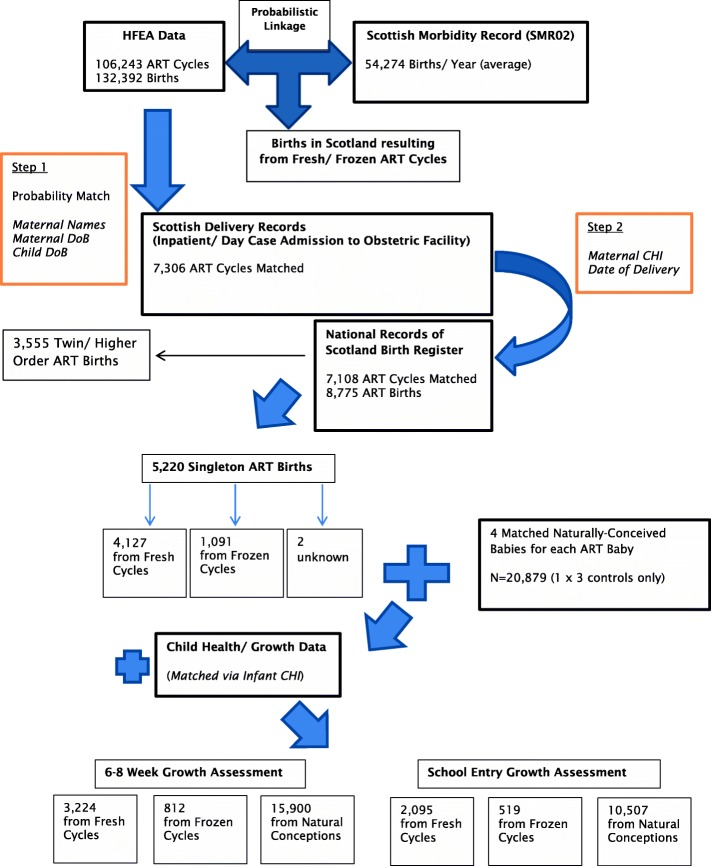
Data linkage steps and resultant ART cycle/birth numbers

A cohort of ART data from the Human Fertilisation and Embryology Authority (HFEA) register was linked to a series of routinely collected birth and child health records held by the NHS National Services Scotland (NHS NSS) (Fig. 1).

#### HFEA Assisted Reproductive Technologies Register

The UK has a uniquely comprehensive record of all women who have had ART since August 1991, when it has been compulsory for every UK fertility clinic to report details of all treatments to the HFEA. Up to October 2009, data on 289,000 women and 110,000 children born after treatment has been collected. Data on treatment cycles after October 1, 2009, was not included as, from this time, those undergoing ART were asked to give “consent to disclosure” of identifying information, enabling their records to be used for research. The data relating to children born prior to April 1997 was not included in the study, as they were over the age of 16 at the time of data release and we took a cautious approach as to whether consent had been provided. The HFEA register does not contain a single unique patient identifier, but does contain names and dates of birth.

#### Scottish Morbidity Record—SMR02

The SMR02 is primarily a source of data on obstetric outcomes, collated from hospital administration records following a woman’s episode of care in an obstetric facility (inpatient and day cases, but not home births). Birth weight, gestational age, Apgar score, occipital-frontal circumference (OFC), length, weight and admission to a neonatal intensive care unit (NICU) have been recorded consistently over time. There is also data on maternal lifestyle with self-reported smoking being consistently recorded. The child’s Community Health Index (CHI) number allows linkage to child data in other datasets.

#### Scottish child health programme

Data on child growth is available from a number of routine screening programmes. The data consistently collected at 6–8 weeks and on entry to school (generally collected between 4 and 7 years) comprise height (length and OFC at 6–8 weeks), weight, body mass index (BMI) and read-coded health concerns.

### Data linkage

In the absence of a unique identifier, HFEA and SMR02 data were linked using probabilistic matching based on maternal names and maternal/child dates-of-birth as described in Additional file 1.

### Statistical analyses

Deprivation was assessed from quintiles of the 2012 Scottish Index of Multiple Deprivation [19]. Maternal smoking history was self-reported.

Birth weight was adjusted for gestational age, gender and (when available) parity (1st baby vs. > 1st baby) using the GROW formula [20].

Growth velocities were defined as the average change in weight per week (between birth and 6–8 week measurements) or month (between 6 and 8 weeks and school entry).

Numerical outcomes were modelled using linear regression while NICU admission and sex ratio (ratio of female to male births) were modelled using logistic regression. Bootstrapped standard errors (using 1000 replications) are presented. Details of all the models considered are given in Additional file 1: Table S4.

For analyses comparing ART and naturally conceived infants, the primary covariates were deprivation, smoking status, maternal age (fitted as a quadratic function) and an indicator of whether the embryos were from fresh or frozen transfer cycles, plus the matching variables, year and month of delivery and gender. For within ART analyses, we additionally included paternal age (as a quadratic function), use of ICSI, number of previous ART cycles and infertility causes (tubal, endometrial, ovulatory, male and idiopathic as binary indicators) plus treatment centre and year of treatment. As data relating to embryo culture time and number of embryos created were only available on a subset of cases (generally not available for FET cycles), these were not included in the primary analyses, but a secondary analysis including these was conducted. For the analysis of child health programme (CHP) outcomes, we additionally controlled for age at the time of measurement and feed type (breast only, bottle only, breast and bottle) at 10 days post-birth.

In the analysis of NICU admission, the primary analysis was unadjusted for birth weight and gestational age. A secondary analysis was also conducted in which both raw birth weight and gestational age were controlled for.

Using tables of age- and gender-specific L, M and S values [21], we transformed school-entry weight into deciles of age-standardised *z*-scores.

All analyses were conducted using STATA (version 14), within the National Safe Haven of Scotland, managed by NHS NSS.

## Results

### Data linkage and analysis datasets

The linkage process and derivation of the analysis datasets is summarised in Fig. 1.

The resultant dataset comprised 4127 singleton births following fresh ART and 1091 singleton births from FET cycles, along with 20,879 matched NC children. See Additional file 1: Tables S1 and S2 for study characteristics and main outcome variables.

At 6–8 weeks, data was available for 3224 fresh ART births (78%), 812 births following FET (74%) and 15,900 naturally conceived births (76%). The corresponding numbers at school entry were 2095 (51%), 519 (48%) and 10,507 (50%).

The proportions and characteristics of the infants contributing to each of the assessments did not vary between conception groups (Additional file 1: Table S3).

Analyses conducted, outcomes and covariates considered and tables in which corresponding results are presented are summarised in Additional file 1: Table S4.

### Newborn health: ART vs. naturally conceived (NC)

To quantify the impact of ART on newborn health, we first compared all ART children to their NC counterparts. Controlling for deprivation, maternal age and maternal smoking status during pregnancy, there was a significant association between each of birth weight (Table 1), OFC and crown-heel length and ART conception (Additional file 1: Table S5).

**Table 1:** Weight at the three assessment times for fresh ART, frozen ART (FET) and naturally conceived (NC) singleton babies

	Adjusted BW (GROW) *N* = 26,010		Weight at 6/8 weeks *N* = 19,028		Weight at school entry *N* = 11,573	
	Effect size (95% CI) (g)	*P*	Effect size (95% CI) (g)	*P*	Effect size (95% CI) (g)	*P*
Type of conception						
Naturally Conceived	Reference	< 0.001	Reference	< 0.001	Reference	0.340
ART - Fresh Cycle	-93.7 (-110.6, -76.6)		-171 (-200, -143)		-58 (-231, 115)	
ART - Frozen Cycle	57.5 (30.7, 86.5)		1 (-47, 49)		200 (-135, 560)	
Scottish Deprivation Index Quintile						
1 (most deprived)	-44.6 (-64.2, -25.7)	< 0.001	-48 (-80, -17)	0.052	38 (-168, 240)	0.131
2	-25.1 (-44.1, -6.4)		-25 (-57, 4)		229 (42, 430)	
3	-6.8 (-24.3, 10.3)		-32 (-61, 0)		104 (-74, 294)	
4	3.8 (-13.8, 22.0)		-15 (-48, 13)		29 (-150, 201)	
5 (least deprived)	Reference		Reference		Reference	
Smoked during pregnancy	-211.1 (-227.6, -195.2)	< 0.001	-190 (-216, -163)	< 0.001	-40 (-194, 136)	0.638
Maternal age (linear)	41.3 (30.1, 52.2)	< 0.001	-7 (-25, 12)	< 0.001	118 (3, 240)	0.011
Maternal age (quadratic)	-20.8 (-35.6, -8.1)		-52 (-75, -29)		-121 (-266, 17)	
Type of feeding						
Breast only			Reference	< 0.001	Reference	0.125
Bottle only			77 (54, 98)		99 (-31, 229)	
Breast and bottle			-9 (-52, 30)		229 (-25, 501	

Results from multiple linear regression models for weight at the three time points, adjusting for type of conception, Scottish Index of Multiple Deprivation, maternal age and smoking status during pregnancy, feed type at 10 days and the ART-NC matching variables: gender, year and month of delivery and delivery hospital. Full model details are available in Additional file 1: Table S4

Quoted effect sizes represent the difference from the indicated reference category. The effect size for maternal smoking represents the difference between mothers who smoked and those who did not smoke during pregnancy

*P* values are calculated from an omnibus chi-squared test that parameter estimates are simultaneously zero

Note that for ease of interpretation, the effect sizes for the linear and quadratic components of maternal age represent the change in outcome for a 10-year change in maternal age

Compared to NC babies, birth weight was significantly lower in babies born from fresh transfers [-93.7 g; 95% CI (-110.6, -76.6)], but significantly higher from FET [57.5 g; 95% CI (30.7, 86.5)] with an increased incidence of macrosomia (> 4000 g) (17% versus 10% in fresh and 12.6% in NC babies (Additional file 1: Table S2). Similarly, OFC and crown-heel length were smaller in babies born from fresh transfers [-2.87 mm; 95% CI (-3.48, -2.24) and -0.36 cm; 95% CI (-0.50, -0.22) respectively], but larger from FET [1.09 mm; 95% CI (0.06, 2.21) and 0.21 cm; 95% CI (-0.05, 0.47) respectively] (Additional file 1: Table S5).

Compared to NC babies, a significantly shorter gestation was seen in ART babies from both fresh transfers [2.0 days; 95% CI (1.4, 2.5)] and FET [1.2 days; 95% CI (0.3, 2.2)] (Additional file 1: Table S5). This was reflected in a higher rate of pre-term birth (PTB)/very PTB in fresh ART babies (8%/1.3%, compared to 7%/1% and 6%/0.9% in FET and NC babies, respectively: Additional file 1: Table S2). There were no significant differences in sex ratio (Additional file 1: Table S5).

Babies conceived from fresh transfers, but not FET, had a higher rate of admission to NICU [OR = 1.24; 95% CI (1.10, 1.40) for fresh and OR = 1.00; 95% CI (0.80, 1.26) for FET]. However, this effect did not remain when we adjusted for gestation and birth weight (Additional file 1: Table S5).

Fitting similar models for adjusted birth weight, but with time as a linear covariate, revealed no significant temporal trends over the period: NC slope -0.21 g/year; 95%CI (-1.81, 1.57); ART fresh transfers slope 2.59 g/year; 95% CI (-1.12, 6.38); FET slope 0.80 g/year; 95% CI (-6.72, 7.75).

### Newborn health: ART treatment variables

Within the ART cohort, we examined the effect of ART treatment factors on birth outcomes (Table 2; Additional file 1: Table S6), controlling for differences in infertility diagnosis, treatment and parity in addition to those factors considered above.

**Table 2:** Weight at the three assessment times in fresh and frozen cycle ART-conceived singleton babies: association with ART treatment parameters

	Adjusted BW (GROW) *N* = 5071		Weight at 6/8 weeks *N* = 3908		Weight at school entry *N* = 2571	
	Effect size (95% CI) (g)	*P*	Effect size (95% CI) (g)	*P*	Effect size (95% CI) (g)	*P*
Fresh embryo-transfer cycle	Reference	< 0.001	Reference	< 0.001	Reference	0.172
Frozen embryo-transfer cycle	127.4 (95.0, 162.8)		133.3 (75.4, 190.2)		257.8 (-115.5, 650.7)	
Scottish Deprivation Index Quintile						
1 (most deprived)	-60.8 (-111.1, -12.0)	0.218	23.9 (-53.4, 101.9)	0.288	494.1 (-63.4, 1051)	0.502
2	-24.0 (-67.6, 16.4)		53.0 (-12.8, 118.3)		67.7 (-328.9, 464.3)	
3	-11.1 (-50.5, 30.0)		29.8 (-29.8, 92.7)		69.3 (-295.1, 431.7)	
4	-14.8 (-51.0, 18.0)		-16.4 (-74.6, 40.0)		22.2 (-321.9, 323.1)	
5 (least deprived)	Reference		Reference		Reference	
Smoked during pregnancy	-181.5 (-230.6, -133.5)	< 0.001	-117.9 (-197.4, -39.5)	0.004	118.6 (-354.0, 667.8)	0.649
Maternal age (linear)	9.8 (-30.2, 52.3)	0.234	-69.9 (-172.4, 41.2)	0.225	205.9 (-440.8, 840.6)	0.818
Maternal age (quadratic)	53.6 (-6.4, 114.4)		14.0 (-90.6, 110.7)		-157.0 (-800.4, 448.9)	
Paternal age (linear)	4.6 (-26.3, 33.3)	0.681	-32.6 (-80.1, 17.0)	0.423	-19.0 (-306.9, 280.7)	0.750
Paternal age (quadratic)	-11.7 (-38.1, 14.8)		11.2 (-35.3, 58.0)		122.9 (-192.7, 504.1)	
ICSI	-8.5 (-47.6, 31.8)	0.674	27.4 (-27.3, 84.3)	0.347	124.8 (-270.9, 551.2)	0.543
No. previous ART cycles						
0	Reference	0.875	Reference	0.764	Reference	0.598
1	5.1 (-31.0, 39.5)		23.6 (-35.6, 77.6)		195.3 (-149.9, 537.8)	
2	-7.0 (-48.0, 36.3)		-7.4 (-76.1, 61.3)		-18.4 (-427.5, 390.0)	
3 or more	11.0 (-29.6, 50.5)		-5.7 (-75.7, 61.2)		-44.9 (-461.8, 342.2)	
Infertility cause						
Fallopian tubal damage	-14.0 (-58.4, 34.4)	0.555	-34.2 (-112.6, 40.1)	0.370	-210.3 (-610.3, 229.3)	0.344
Endometrial	-18.4 (-65.2, 35.5)	0.482	-71.0 (-148.0, 13.1)	0.074	-470.8 (-915.2, -33.0)	0.042
Ovulatory disorders	-22.1 (-77.1, 33.0)	0.426	-37.8 (-125.7, 50.2)	0.409	-88.8 (-604.9, 408.4)	0.725
Male-factor cause	-13.5 (-52.9, 28.4)	0.527	-28.1 (-90.4, 32.4)	0.364	-12.1 (-375.0, 352.7)	0.949
Idiopathic	-42.8 (-87.3, 5.9)	0.070	-44.2 (-114.6, 35.7)	0.229	-272.0 (-673.0, 173.5)	0.206

Results from multiple linear regression models for weight at the three time points, adjusting for type of ART conception, Scottish Index of Multiple Deprivation, maternal age and smoking status during pregnancy, paternal age, whether or not ICSI was used, the number of previous ART cycles and causes of infertility. Full model details are available in Additional file 1: Table S4

Quoted effect sizes represent the difference from the indicated reference category. The effect size for maternal smoking represents the difference between mothers who smoked and those who did not smoke during pregnancy. The effect size for ICSI represents the difference between ART cycles in which ICSI was used and those in which it was not. The effect size for infertility causes represents the difference between the stated cause being identified within the couple and not being identified

*P* values are calculated from an omnibus chi-squared test that parameter estimates are simultaneously zero

Note that for ease of interpretation, the effect sizes for the linear and quadratic components of maternal/paternal age represent the change in outcome for a 10-year change in maternal/paternal age

The difference in (adjusted) birth weight between babies from FET, compared to fresh transfer, was estimated as 127.4 g [95% CI (95.0, 162.8)]. Both OFC [by 3.6 cm; 95% CI (2.5, 4.7)] and crown-heel length [by 0.5 cm; 95% CI (0.2, 0.8)] were significantly larger in babies born following FET. There were no significant differences in gestational age, sex ratio or NICU admission (Additional file 1: Table S6). No other IVF factors available had a detectable impact on birthweight, OFC, crown-heel length, gestation, sex ratio or NICU admission (Additional file 1: Table S6).

### Infant growth at 6–8 weeks and school entry

**Fig. 2:**
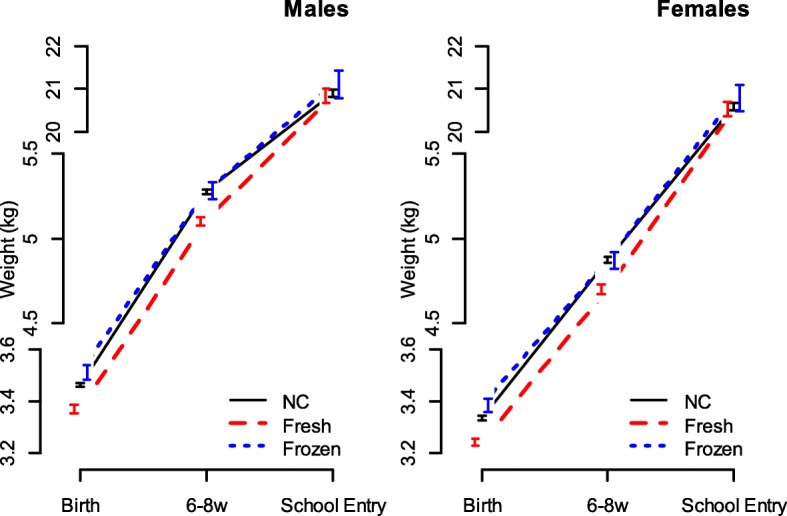
Infant weights at the three assessment points: birth (gestation-adjusted), 6–8 weeks and school entry (5–7 years) for ART-conceived babies following fresh and frozen embryo transfers and naturally conceived controls

Controlling for maternal age, deprivation, smoking and type of feeding, at 6–8 weeks, ART babies from fresh transfers remain smaller than NC babies in respect of their weight [by 171 g; 95% CI (143, 200)], OFC [by 2.41 mm; 95% CI (1.87, 3.05)] and length [by 0.39 cm; 95% CI (0.27, 0.52)], but those from FET show no differences (Fig. 2; Additional file 1: Table S7).

By school entry, we can no longer detect any significant differences in weight between ART and NC children (Fig. 2; Additional file 1: Table S8). However, ART children are very slightly taller [fresh by 0.28 cm; 95% CI (-0.01, 0.53): frozen by 0.68 cm; 95% CI (0.25, 1.13)] and show lower BMI [fresh by -0.12; 95% CI (-0.21, -0.03): frozen by -0.04; 95% CI (-0.022, 0.14)] (Additional file 1: Table S8).

Analysis of ART factors (Additional file 1: Table S9) shows that babies from FET cycles are significantly heavier at 6–8 weeks than those from fresh cycles by 133 g [95% CI (75, 190)] and they tend to remain heavier at school entry, although significance is lost [by 258 g; 95% CI (-116, 651)]. FET babies are significantly taller at school entry [by 0.56 cm; 95% CI (0.07, 1.11)], but no significant difference was seen in BMI. No significant difference in the growth parameters was seen with any of the other ART treatment/diagnosis factors.

### Infant growth rates from birth to 6–8 weeks and school entry

We have restricted this analysis to a consideration of weight, which we have for all three time points.

Table 3 shows the linear regression models for growth rate, controlling for gender, deprivation, maternal smoking and age and type of feeding. Babies born from fresh ART grew significantly faster between birth and 6–8 weeks than NC babies, by 7.2 g/week [95% CI (2.1, 12.7)], while babies born from FET were not significantly different. ART-conceived babies grew at a greater rate than NC babies over the period 6–8 weeks to school entry [Fresh vs. NC, 0.25 g/week, 95% CI (-0.34, 0.88); FET vs. NC, 0.74 g/week, 95% CI (-0.39, 1.90)].

**Table 3:** Growth rates of singleton births from fresh ART transfers, frozen transfers (FET) and naturally conceived (NC) babies between birth and 6–8 weeks, and 6–8 weeks and primary school entry

	Birth/6–8 weeks		6–8 weeks/school entry	
	Coefficient (95% CI)	*P*	Coefficient (95% CI)	*P*
*Constant* (growth rate for a NC baby with ‘Reference’ characteristics)	254.8 (250.5, 259.1)		54.4 (53.9, 54.9)	
*Type of conception*				
Naturally conceived	Reference	0.023	Reference	0.363
ART—fresh cycles	7.2 (2.1, 12.7)		0.25 (-0.34, 0.88)	
ART—frozen cycles	4.9 (-3.5, 13.9)		0.74 (-0.39, 1.90)	
*Gender*				
Male	Reference	< 0.001	Reference	0.025
Female	-46.2 (-49.4, -42.9)		0.48 (0.06, 0.89)	
*IMD Quintile*				
1 (most deprived)	8.2 (3.1, 13.3)	0.026	0.46 (-0.23, 1.20)	0.102
2	6.7 (1.4, 11.9)		0.90 (0.23, 1.58)	
3	6.8 (1.5, 12.4)		0.52 (-0.10, 1.20)	
4	5.4 (-0.1, 10.9)		0.21 (-0.41, 0.80)	
5 (least deprived)	Reference		Reference	
*Smoked during pregnancy*				
No	Reference	< 0.001	Reference	0.241
Yes	19.6 (14.9, 24.5)		0.36 (-0.22, 0.95)	
*Maternal age*				
(Linear term)	2.9 (-0.2, 6.4)	0.076	0.42 (0.01, 0.84)	0.032
(Quadratic term)	-2.4 (-6.7, 1.8)		-0.29 (-0.76, 0.19)	
*Feed type at 10 days*				
Breast	Reference	< 0.001	Reference	0.236
Bottle	22.8 (18.7, 26.5)		0.12 (-0.35, 0.57)	
Breast and bottle	11.7 (4.6, 18.4)		0.78 (-0.10, 1.66)	

Results from multiple linear regression models for growth (change in weight) between birth and 6–8 weeks (average weekly growth rate) and 6–8 weeks and primary school entry (average monthly growth rate), adjusting for type of conception, Scottish Index of Multiple Deprivation (IMD), maternal age and smoking status during pregnancy, feed type at 10 days and the ART-NC matching variables: gender, year and month of delivery and delivery hospital. Full model details are available in Additional file 1: Table S4

Quoted effect sizes represent the difference from the indicated reference category. The ‘Constant’ coefficient represents the average rate of growth for a naturally conceived boy, breast-fed at 10 days by a non-smoking mother who resided in an area belonging to the least deprived quintile

*P* values are calculated from an omnibus chi-squared test that parameter estimates are simultaneously zero

Note that for ease of interpretation, the effect sizes for the linear and quadratic components of maternal age represent the change in outcome for a 10-year change in maternal age

No ART-related factors were significantly associated with either growth rate (Additional file 1: Table S10).

### Catch-up growth

Of particular interest is the proportion of infants who show accelerated (‘catch-up’) or decelerated (‘catch-down’) growth (weight) from birth to school entry. In Table 4, we show the numbers who crossed more than one decile in the age/gender adjusted growth charts in the three conception groups. Significantly, more fresh ART babies show catch-up growth from birth to school entry, compared to NC (27% vs. 24%; *P* = 0.004), but this is not seen in just the smaller babies (lower 3 deciles). Significantly, less catch-down growth is seen in the fresh ART cohort (vs. NC, 23% vs. 27%; *P* = 0.002) and this is also seen when considering only the largest babies (43% vs. 48%; *P* = 0.014). FET babies generally show more similar rates of catch-up and catch-down growth to NC babies than to fresh ART babies.

**Table 4:** Catch-up and catch-down growth between birth and school entry

	Naturally conceived	Fresh ART		Frozen ART (FET)	
	*n* (%)	*n* (%)	*P*	*n* (%)	*P*
All babies: catch-up	2491/10500 (23.7%)	559/2095 (26.7%)	0.004	104/519 (20.0%)	0.053
All babies: catch-down	2834/10500 (27.0%)	483/2095 (23.1%)	0.002	155/519 (29.9%)	0.15
Small babies: catch-up	1237/2238 (55.3%)	283/492 (57.5%)	0.36	43/75 (57.3%)	0.72
Large babies: catch-down	1928/4015 (48.0%)	306/711 (43.0%)	0.014	116/240 (48.3%)	0.92

Catch up/down is defined as a change of more than one decile in the standardised growth charts (between birth and primary school entry)

*P* values are from a *χ*^2^ test comparing, separately, ART fresh with naturally conceived children and FET with naturally conceived children

Numbers and proportions are shown for all children experiencing catch-up (first row) and catch-down (second row) growth: then catch-up growth for those babies who were small at birth (defined here as 1st–3rd deciles - third row) and catch-down growth for those babies who were large at birth (defined here as 8th–10th deciles - fourth row)

## Discussion

Birth weight and early child growth are important predictors of long-term CMD risk [6]. Although it is well established that ART children are at increased risk of low birth weight, there is little understanding of the causal factors in the ART process and there have been no large studies of postnatal growth in these children. Using the UK ART data register held by the HFEA, linked to maternity and child health data, we conducted a large-scale analysis of more than 5000 singleton ART children. Our key findings are that not only do ART babies born from fresh embryo transfers have lower weights, head circumference and length at birth than NC children, they grow more quickly and catch up by school age. Babies from FET cycles, by contrast, have greater weight, head circumference and length at birth and show similar growth to NC children.

The major strength of this study is the registry-based national cohort design. The loss to follow-up is a concern, although we believe most of this loss is administrative and we could detect no strong biases in those with and without data. The data on NC infants did not include parity and, therefore, we were not able to include this in the birth weight adjustment. It is likely that more NC babies were born to parous women than ART-conceived babies, which would lead to a small, but unquantifiable, over-estimation of birth weight in this group, in turn reducing the effect in ART-Fresh cycles while increasing the effect in FET cycles. Within-ART comparisons were parity-adjusted. Postnatal growth estimates for both ART-conceived and NC children are based on changes and are therefore largely unaffected by parity.

Newborn infants conceived by fresh embryo transfer were smaller than NC infants, with significant differences in birth weight, length and OFC. Some infants were small because they were born early, at gestational ages of < 37 or < 32 weeks, and there were more admissions to the NICU, probably due to conditions associated with prematurity. Other infants were small for gestational age (SGA), which may be due to a slowing down of intrauterine growth, implicating a reduction in placental transfer of nutrients. FET infants by comparison had a higher birth weight, length and OFC than NC infants. The birth weight findings are consistent with previous studies [2, 4, 22], and the weight differences are remarkably consistent across studies, e.g. in a recent prospective RCT in China, FET babies were 161 g larger than fresh ART babies [16] and in a large Japanese registry study, FET babies were 91 g larger than fresh ART, and 40 g larger than NC babies [23], very similar findings to ours.

At 6 to 8 weeks of age, the weight, length and OFC of the FET infants and NC infants were similar, while weight, length and OFC in the fresh ART-conceived infants remained smaller. At school entry, weight, length and BMI in the three study groups were similar, showing that low birth weight fresh ART infants, when free from the constraints on intrauterine growth, resume their growth trajectory postnatally and attempt to achieve their genetic potential by catch-up growth. OFC measurements were not available; however, brain growth and somatic growth are likely to be in harmony. The BMI values at school entry were consistent with those reported previously at a similar age.

Previous studies of ART child growth have been small (≤ 500 children) and unable to adequately control for confounding factors, and show conflicting results [12]. In three small European cohort studies, children born from intra-cytoplasmic sperm injection (ICSI) were found to be lighter than their target weight (for age and height) at age 3 years, but not at age 5 years [12]. In a longitudinal study of approximately 200 ART children, they had lower weight at birth and 3 months, with no difference in early growth rate (velocity) compared to a subfertile NC control group; however, ART children then showed greater growth velocity from 3 months to 12 months, followed by similar childhood growth rates to age 3 [13]. Small studies of IVF and ICSI children measured at birth and up to age 18 months in Taiwan [24], 3 years in the USA [25], 4 years in the Netherlands [26] and 10–12 years in the UK [27] showed few differences in growth [12]. Data from a UK child screening programme [28] showed that ART children were less likely to be overweight at 5 years old than controls, with significantly lower BMIs at 7 years of age than controls consisting of births after normal conception or to subfertile couples. Green et al. [29] controlled for embryo freezing as a confounding factor, and showed that fresh transfer IVF children, while lower birthweight than FET or NC children, did not show catch up weight growth in childhood; however, ART conceived girls were taller than matched controls after fresh IVF and also FET [29, 30]. Our data extend this current literature significantly by virtue of the large sample size and careful adjustment for confounding factors, by use of accurate growth data recorded by maternity units including length and OFC in addition to birth weight and, most importantly, by following growth of children to age of school entry.

It is currently unknown what alters fetal and child growth trajectories for children conceived through ART; however, it is noteworthy that the magnitude of impact of ART on birth weight is nearly as great as that of maternal smoking in pregnancy. Catch-up or catch-down growth (centile crossing) after birth are both significant predictors of long-term disease risk. Most LBW children show catch-up growth in the first few months, with accelerated growth associated with increased risk of obesity and CMD risk [11, 31, 32]. Our study shows that fresh ART babies grow faster, from birth, over the first few weeks, than their NC counterparts with further catch-up growth then seen in fresh ART children by school entry age. This is associated, in NC children, with increased risk of obesity and CMD in adulthood [10–12, 33, 34] and, in ART children, with greater weight gain during childhood, associated with increased cardiovascular risks [13]. Reassuringly, however, catch-up was not seen in the smallest fresh ART birth weight centiles in our study, whereas the increased rate of catch-down growth observed was strongest for the largest birth weight centile babies.

Our major finding that children born from FET cycles differ significantly in birth weight and child growth from their fresh ART counterparts, strongly implicates some aspect of the ART treatment process itself rather than parental subfertility, as does the animal literature showing that adverse outcomes can be phenocopied in fertile mice subjected to ART [9]. However, the mechanisms involved are unknown. One plausible hypothesis is that the uterine environment is hormonally dysregulated following ovarian hyper-stimulation, such that in fresh cycles fetal growth is restricted due to impaired placental function [5, 35]. Alternatively, the ART environment may act via a direct epigenetic effect on the embryo; however, this would predict that the impaired fetal growth trajectory would be continued into postnatal life, and so is not supported by our data. Fetal growth in FET cycles may also be affected by some aspect of the embryo cryopreservation process, consistent with our previous finding that frozen/thawed embryos show altered gene expression [5] and our finding (and from others [23]) that FET babies have a consistently slightly higher birth weight compared to NC babies.

Other than the effects of embryo cryopreservation/FET, we failed to detect any significant associations between newborn or child weight, length and OFC and other ART patient or treatment factors available. However, the HFEA database lacks information on factors such as the type of culture medium embryos were grown in, an important omission in light of recent evidence that culture medium is associated with altered embryo gene expression [15], epigenetics [9] and fetal and postnatal growth following ART [15], resulting in calls for action from professional societies and leading scientific journals [5].

## Conclusions

These longitudinal growth data from a national cohort of more than 5000 ART children identify an increased risk profile for non-communicable disease in later life. ART treatments are constantly evolving, and these studies will need to be replicated in more recent cohorts to capture changes in technology and practice. There is clearly a need to further the research agenda in this area and to take steps to reduce risks from treatment. We call for more detailed studies of the impact of modifiable aspects of IVF on early child growth to reduce risk to the next generation of ART children.

## Additional file

Additional file 1: Supplementary Descriptive Text and Tables. (DOCX 124 kb)

## Abbreviations

95% CI: (95%) confidence interval
(L)BW: (Low) birth weight
(NHS) NSS: (National Health Service) National Services Scotland
(S)GA: (Small for) gestational age
ABW: Adjusted birth weight
ART: Assisted reproductive technology/technologies
BMI: Body mass index
CHI: Community Health Index
CHP: Child health programme
CMD: Cardio-metabolic disease
DOHaD: Developmental Origins of Health and Disease
FET: Frozen embryo transfer
GROW (formula): Gestation-related optimal weight
HFEA: Human Fertilisation and Embryology Authority
ICSI: Intra-cytoplasmic sperm injection
IMD: Index of Multiple Deprivation
IQR: Inter-quartile range
IVF: In vitro fertilisation
NC: Naturally conceived
NICU: Neonatal intensive care unit
OFC: Occipital frontal circumference
PSE: Primary school entry
PTB: Pre-term birth
RCT: Randomised controlled trial
SD: Standard deviation
SMR(02): Scottish Morbidity Record
