# Supplementary material for: The growth of assisted reproductive treatment-conceived children from birth to 5 years: a national cohort study
Source: BMC Med. 2018 Nov 28;16:224. doi: 10.1186/s12916-018-1203-7 (PMC6260690; doi:10.1186/s12916-018-1203-7)
Supplement: Supplementary file 1 — Supplementary Descriptive Text and Tables. (DOCX 124 kb) [file 12916_2018_1203_MOESM1_ESM.docx]

# Additional file 1

# The growth of Assisted Reproductive Treatment-conceived children from birth to 5 years: A national cohort study

### Mark Hann PhD ^1^, Stephen A. Roberts PhD ^1^, Stephen W D’Souza FRCPCH ^2^, Peter Clayton MD ^3^, Nick Macklon MD ^4, 5^, Daniel R. Brison PhD ^2, 6^

^1^ Centre for Biostatistics, Division of Population Health, Health Services Research and Primary Care, School of Health Sciences, Faculty of Biology, Medicine and Health, University of Manchester, Oxford Road, Manchester M13 9PT.

^2^ Maternal and Fetal Health Research Centre, and ^3^ Child Health & Paediatric Endocrinology, Division of Developmental Biology & Medicine, School of Medicine, Faculty of Biology, Medicine and Health, University of Manchester, Manchester Academic Health Sciences Centre, Oxford Road, Manchester M13 9PT.

^4^ Human Development and Health Academic Unit, Faculty of Medicine, University of Southampton, Southampton. SO16 6YD.

^5^ Department of Obstetrics and Gynaecology, Princess Anne Hospital, Coxford Road, Southampton. SO16 5YA.

^6^ Department of Reproductive Medicine, Saint Mary's Hospital, Manchester University NHS Foundation Trust, Manchester Academic Health Sciences Centre, Oxford Road, Manchester M13 9PT.

The following material is supportive of the Methods and Results contained in “The growth of Assisted Reproductive Treatment-conceived children from birth to 5 years: A national cohort study” by Mark Hann et al.

## Contents

Details of Data Linkage

Results of Data Linkage

Analyses

**References:** References cited in the supplement

**Table S1:** Characteristics of the Mothers, Fathers and Babies, and the ART Treatments undertaken, in the analysis datasets.

**Table S2**. Summary Statistics for Outcome Variables.

**Table S3**: Available Data by Conception-Type, Index of Multiple Deprivation (IMD) Quintile, Maternal Smoking Status and Maternal Age at Birth.

**Table S4**: Details of Statistical Models.

**Table S5**: Birth Outcomes for Fresh ART, Frozen ART (FET) and Naturally-Conceived Singleton Babies.

**Table S6**: Birth Outcomes in Fresh and Frozen Cycle ART-Conceived Singleton Babies: Association with ART treatment parameters.

**Table S7:** Infant weight, occipital frontal circumference (OFC) and length at 6-8 weeks in ART and Naturally-Conceived Singleton Babies.

**Table S8:** Weight, Height and Body Mass Index (BMI) at Primary School entry in ART and Naturally-Conceived Singleton Babies.

**Table S9**: Infant Weight at 6-8 weeks and Weight, Height and Body Mass Index (BMI) at Primary School entry in Fresh and Frozen Cycle ART-Conceived Singleton Babies: Association with ART treatment parameters.

**Table S10**: Growth Rates of Singleton Births from Fresh and Frozen (FET) ART Transfers between Birth and 6-8 Weeks and 6-8 Weeks and Primary School Entry: Association with ART treatment parameters.

## Details of Data Linkage

In the absence of a unique identifier, HFEA and SMR02 data were linked using probabilistic matching based on maternal names and maternal/child dates-of-birth. Corresponding data fields from the different sources were compared and an indication of how closely they match was derived. Given these are weaker identifiers than, for example, a unique identifier specific to an individual, some of the effects that we report here may be under-estimates.

Given the size of the data-sets to hand, it was unrealistic to be able to assess the probability of a match amongst every pair of records. Therefore, the identifying items were first split into, and compared within, two ‘blocks’: Soundex/ NYSIIS ^1^ encoded, first initial and sex (Block A) and all elements of date-of-birth (day, month and year - Block B). Records were not further compared if they disagreed on one or more of the items in Block A and also disagreed on one or more of the items in Block B. Whilst it was possible that two records belonging to the same person disagreed on, for example, first initial and date of birth, previous linkage attempts with SMR02 data have shown that the proportion of true links lost because of ‘blocking’ was less than 0.5% ^2^.

Once a potential match had been established, an assessment of the level of agreement (and disagreement) was made. In its simplest form, every time an item of identifying information was the same on the two records, the probability that they applied to the same person increased. Conversely, every time that an item of identifying information differed, the probability that they applied to the same person decreased. Automated computer algorithms ^2,3^ determine whether the two records from the two different data sources are ultimately classified as ‘truly linked’ or ‘truly unlinked’.

Child Health Programme (CHP) data was linked using the child’s CHI Number, which creates a much stronger link.

For every ART singleton baby, 4 naturally conceived (NC) singleton babies of the same sex, year and month of delivery and delivered at the same hospital were identified.

## Results of Data Linkage

In all, 7,108 cycles (resulting in 8,775 babies) were matched, which compares well with the number of cycles (7,700) and babies (9,000) estimated from the place of birth recorded in the HFEA register. Data loss could be attributed to ‘false links’ (resulting in a non-Scottish birth), failure to find a matching birth record or missing CHI numbers.

Of the 8,775 babies, 3,555 were identified as either twin births or births of a higher order, with 5,220 identified as singleton births, of which 4,127 were born as a result of fresh ART, 1,091 as a result of FET and 2 were unknown.

## Analyses

The analyses here relate to singleton pregnancies resulting in a singleton live birth. HFEA data records the number of fetal hearts detected, whilst the Scottish Birth Record records the number of children per pregnancy: both were required to be 1.

Importantly, we observed that in 315 of 5,209 cases (6%) where data was available from both sources, the patient-reported birth weights recorded on the HFEA register were discrepant from those in SMR02 (used in the analyses) by more than 100g with no tendency for either data source to consistently report heavier or lighter birth weights.

**References**

1. Newcombe HB. Handbook of Record Linkage, OUP: New York, 1988. New York: OUP; 1988.

2. Kendrick S, Clarke J. The Scottish Record Linkage System. *Health Bull (Edinb)* 1993; **51**(2): 72-9.

3. Fellegi IP, Sunter AB. A Theory for Record Linkage. *J Am Stat Assoc* 1969; **64**(328): 1183-210.

**Table S1:** Characteristics of the Mothers, Fathers and Babies, and the ART Treatments undertaken, in the analysis datasets.

|  |  | **ART Fresh** | **ART Frozen (FET)** | **Naturally Conceived** |
| --- | --- | --- | --- | --- |
|  | N | 4,127 | 1,091 | 20,879 |
| **Child** | | | | |
| Gestation at birth (weeks) | Median (IQR) | 39 (38, 40) | 39 (38, 40) | 40 (39, 41) |
|  | Female  Male | 2,026 (49.09%)  2,101 (50.91%) | 536 (49.13%)  555 (50.87%) | 10,266 (49.17%)  10,612 (50.83%) |
| Age at first assessment (days) | Median (IQR) | 52 (45, 60)  [N=3,283] | 52 (45, 59)  [N=827] | 54 (45,61)  [N=16,184] |
| Age at pre-school assessment (months) | Median (IQR) | 66.9 (63.4, 69.9)  [N=2,095] | 66.7 (64.0, 70.1)  [N=519 | 66.6 (63.4, 69.8)  [N=10,509] |
| **Mother** | | | | |
| Previous Births | 0  1  2  3+ | 3,875 (93.89%)  244 (5.91%)  6 (0.15%)  2 (0.05%) | 804 (73.69%)  274 (25.11%)  13 (1.19%)  0 | Data not Available |
| Smoked During Pregnancy | Y  N  Unknown | 357 (8.65%)  3,480 (84.32%)  290 (7.03%) | 108 (9.90%)  908 (83.23%)  75 (6.87%) | 4,694 (22.48%)  14,678 (70.30%)  1,507 (7.22%) |
| Index of Multiple Deprivation Quintile | 1 (Most Deprived)  2  3  4  5 (Least Deprived) | 449 (10.90%)  574 (13.94%)  753 (18.28%)  1,004 (24.37%)  1,339 (32.51%) | 113 (10.39%)  135 (12.41%)  204 (18.75%)  261 (23.99%)  375 (34.47%) | 5,356 (25.72%)  4,139 (19.87%)  3,851 (18.49%)  3,756 (18.03%)  3,726 (17.89%) |
| Age at child’s birth | Mean (SD)  Median (IQR) | 34.5 (3.9)  35 (32, 37) | 34.6 (4.2)  35 (32, 38) | 28.6 (6.0)  29 (24, 33) |
| Cause of Infertility  (Couples may have multiple causes) | Fallopian Tubal blockage/damage Endometriosis Ovulatory Disorders  Male Factor  Unknown | 1,000 (24.80%)  368 (9.13%)  300 (7.44%)  2,047 (50.77%)  1,242 (30.80%) | 297 (28.02%)  71 (6.70%)  101 (9.53%)  534 (50.38%)  311 (29.34%) | Not Applicable |
| Previous ART Cycles | 0  1  2  3+ | 2,247 (54.45%)  865 (20.96%)  484 (11.73%)  531 (12.87%) | 12 (1.10%)  453 (41.52%)  263 (24.11%)  363 (33.27%) | Not Applicable |
| **Father** | | | | |
| Age at child’s birth | Mean (SD)  Median (IQR) | 36.3 (5.5)  36 (33, 39) | 36.7 (5.7)  36 (33, 40) | Data not Available |
| **ART treatment** | | | | |
| Culture Time | <2 Days  2 Days  3 Days  >3 Days | 23 (0.65%)  2,393 (67.94%)  1,047 (29.73%)  59 (1.68%) | Data not Available | Not Applicable |
| Intra-Cytoplasmic Sperm Injection |  | 1,599 (38.74%) | 289 (26.49%) | Not Applicable |
| Embryos Created | 1  2  3  4/5  6-8  9-12  >12 | 107 (3.03%)  282 (7.98%)  377 (10.66%)  863 (24.41%)  984 (27.84%)  695 (19.66%)  227 (6.42%) | Data not Available | Not Applicable |
| Non-Scottish  Treatment Centre |  | 143 (3.46%) | 16 (1.47%) | Not Applicable |

The N for ‘Age at first assessment’ and ‘Age at pre-school assessment’ is based on the number completing this assessment (see Figure 1).

**Table S2**. Summary Statistics for Outcome Variables.

|  |  | **ART Fresh** | **ART Frozen (FET)** | **Naturally Conceived** |
| --- | --- | --- | --- | --- |
| **Birth Outcomes** | **N** | **4,127** | **1,091** | **20,879** |
| Raw Birth Weight (g) | N  Mean (SD) | 4,127  3,317.27 (595.71) | 1,091  3,476.33 (586.30) | 20,871  3,397.38 (580.36) |
| Boys | N  Mean (SD) | 2,101  3,368.63 (604.16) | 555  3,537.60 (589.27) | 10,608  3,454.67 (595.58) |
| Girls | N  Mean (SD) | 2,026  3,264.02 (582.20) | 536  3,412.89 (576.92) | 10,262  3,338.28 (557.97) |
| Raw Birth Weight:  >4,000g  <2,500g  <1,500g | N (%)  N (%)  N (%) | 415 (10.06)  294 ( 7.12)  50 ( 1.21) | 188 (17.23)  51 ( 4.67)  8 ( 0.73) | 2,631 (12.60)  1,190 ( 5.70)  162 ( 0.78) |
| Adjusted Birth Weight (g) | N  Mean (SD), adjusted for:  Gestation/Gender  Gestation/Gender/ Parity | 4,127  3,491.49 (474.53)  3,484.81 (473.01) | 1,091  3,641.74 (471.57)  3,612.84 (466.50) | 20,854  3,522.76 (479.55)  Parity not recorded |
| Occipital Frontal Circumference (OFC) (mm) | N  Mean (SD) | 3,331  345.58 (15.98) | 901  349.45 (16.95) | 16,991  346.99 (15.48) |
| Boys | N  Mean (SD) | 1,705  348.14 (15.20) | 452  353.29 (18.53) | 8,518  350.00 (15.55) |
| Girls | N  Mean (SD) | 1,626  342.90 (16.33) | 449  345.59 (14.21) | 8,473  343.96 (14.80) |
| Crown-Heel Length (cm) | N  Mean (SD) | 2,413  50.73 (3.07) | 661  51.26 (3.26) | 12,401  50.84 (3.05) |
| Boys | N  Mean (SD) | 1,226  51.09 (3.03) | 332  51.61 (3.37) | 6,258  51.20 (3.04) |
| Girls | N  Mean (SD) | 1,187  50.34 (3.08) | 329  50.92 (3.12) | 6,143  50.48 (3.02) |
| Neonatal Intensive Care Unit Admission | Admitted/ N  (%) | 422/ 4,064  (10.38) | 95/ 1,078  (8.81) | 1,826/ 20,525 (8.90) |
| Gestation (weeks) | N  Mean (SD) | 4,127  39.05 (2.13) | 1,091  39.15 (2.02) | 20,863  39.32 (1.92) |
| Gestational Age:  <37 weeks  <32 weeks | N (%)  N (%) | 334 (8.09)  54 (1.31) | 78 (7.15)  11 (1.01) | 1,258 (6.03)  178 (0.85) |
| Sex Ratio | N of Boys  N of Girls  (Boys per 100 Girls) | 2,101  2,026  103.70 | 555  536  103.54 | 10,612  10,266  103.37 |
| **6-8 Week Assessment** | **N** | **3,224** | **812** | **15,900** |
| Weight (Kg) | N  Mean (SD) | 3,208  4.92 (0.72) | 809  5.08 (0.72) | 15,831  5.08 (0.74) |
| Boys | N  Mean (SD) | 1,626  5.12 (0.72) | 405  5.28 (0.71) | 8,007  5.29 (0.75) |
| Girls | N  Mean (SD) | 1,582  4.72 (0.66) | 404  4.88 (0.66) | 7,824  4.87 (0.67) |
| OFC (mm) | N  Mean (SD) | 3,169  386.29 (15.24) | 793  388.64 (15.78) | 15,640  387.98 (15.64) |
| Boys | N  Mean (SD) | 1,604  391.21 (14.57) | 402  393.61 (16.17) | 7,919  392.82 (15.35) |
| Girls | N  Mean (SD) | 1,565  381.24 (14.22) | 391  383.53 (13.61) | 7,721  383.01 (14.31) |
| Length (cm) | N  Mean (SD) | 3,166  56.68 (3.08) | 803  57.13 (2.94) | 15,639  57.01 (3.04) |
| Boys | N  Mean (SD) | 1,608  57.32 (3.06) | 409  57.74 (3.15) | 7,920  57.66 (3.04) |
| Girls | N  Mean (SD) | 1,558  56.03 (2.98) | 394  56.50 (2.56) | 7,719  56.35 (2.88) |
| **School Entry Assessment** | **N** | **2,095** | **519** | **10,507** |
| Weight (Kg) | N  Mean (SD) | 2,095  20.79 (3.29) | 519  21.05 (3.53) | 10,496  20.72 (3.37) |
| Boys | N  Mean (SD) | 1,066  20.98 (3.19) | 264  21.32 (3.61) | 5,324  20.95 (3.39) |
| Girls | N  Mean (SD) | 1,029  20.60 (3.39) | 255  20.77 (3.42) | 5,172  20.49 (3.34) |
| OFC (mm) |  | Not Recorded | | |
| Height (cm) | N  Mean (SD) | 2,093  113.83 (5.31) | 519  114.19 (5.01) | 10,490  113.02 (5.33) |
| Boys | N  Mean (SD) | 1,065  114.43 (5.34) | 264  114.96 (5.17) | 5,321  113.70 (5.32) |
| Girls | N  Mean (SD) | 1,028  113.21 (5.20) | 255  113.40 (4.71) | 5,169  112.31 (5.24) |
| Body Mass Index (Kg/m^2^) | N  Mean (SD) | 2,094  15.98 (1.69) | 519  16.07 (1.79) | 10,484  16.15 (1.77) |
| Boys | N  Mean (SD) | 1,065  15.97 (1.58) | 264  16.05 (1.75) | 5,318  16.13 (1.75) |
| Girls | N  Mean (SD) | 1,029  15.99 (1.81) | 255  16.08 (1.84) | 5,166  16.17 (1.78) |

**Table S3**: Available Data by Conception-Type, Index of Multiple Deprivation (IMD) Quintile, Maternal Smoking Status and Maternal Age at Birth.

| N (% - of N at Birth) |  | Birth |  |  | 6-8 Weeks |  |  | School Entry |  |
| --- | --- | --- | --- | --- | --- | --- | --- | --- | --- |
|  | Natural Conception | ART Fresh | FET | Natural Conception | ART Fresh | FET | Natural Conception | ART Fresh | FET |
| IMD Quintile | | | | | | | | | |
| 1 (Most Deprived) | 5,356 | 449 | 113 | 4,505 (84.1) | 383 (85.3) | 101 (89.4) | 2,714 (50.7) | 229 (51.0) | 68 (60.2) |
| 2 | 4,139 | 574 | 135 | 3,252 (78.6) | 475 (82.8) | 110 (81.5) | 2,207 (53.3) | 317 (55.2) | 74 (54.8) |
| 3 | 3,851 | 753 | 204 | 2,829 (73.5) | 579 (76.9) | 148 (72.5) | 1,931 (50.1) | 407 (54.1) | 100 (49.0) |
| 4 | 3,756 | 1,004 | 261 | 2,680 (71.4) | 775 (77.2) | 189 (72.4) | 1,813 (48.3) | 499 (49.7) | 125 (47.9) |
| 5 (Least Deprived) | 3,726 | 1,339 | 375 | 2,593 (69.6) | 1,006 (75.1) | 263 (70.1) | 1,816 (48.7) | 640 (47.8) | 152 (40.5) |
| Unknown | 51 | 8 | 3 | 41 (80.4) | 6 (75.0) | 1 (33.3) | 26 (51.0) | 3 (37.5) | 0 (0.0) |
| Maternal Smoking Status | | | | | | | | | |
| Yes | 14,678 | 3,480 | 908 | 11,106 (75.7) | 2,696 (77.5) | 671 (73.9) | 7,218 (49.2) | 1,716 (49.3) | 425 (46.8) |
| No | 4,694 | 357 | 108 | 3,532 (75.2) | 281 (78.7) | 77 (71.3) | 2,439 (52.0) | 192 (53.8) | 50 (46.3) |
| Unknown | 1,507 | 290 | 75 | 1,262 (83.7) | 247 (85.2) | 64 (85.3) | 850 (56.4) | 187 (64.5) | 44 (58.7) |
| Maternal Age at Birth | | | | | | | | | |
| Mean (SD) | 28.6 (6.0) | 34.5 (3.9) | 34.6 (4.2) | 28.7 (6.1) | 34.5 (3.8) | 34.6 (4.3) | 28.6 (6.1) | 34.5 (3.8) | 34.5 (4.1) |
| Median (IQR) | 29 (24, 33) | 35 (32, 37) | 35 (32, 38) | 29 (24, 33) | 35 (32, 37) | 35 (32, 38) | 29 (24, 33) | 35 (32, 37) | 35 (32, 37) |
| Total | 20,879 | 4,127 | 1,091 | 15,900 (76.2) | 3,224 (78.1) | 812 (74.4) | 10,507 (50.3) | 2,095 (50.8) | 519 (47.6) |

Figures in parentheses denote the percentage of data at birth that is now available at 6-8 weeks/ school entry.

**Table S4**: Details of Statistical Models.

| **Dataset** | | **Outcome Variable** | **Adjustment**  **(if any)** | **Independent Variables** | **Table** | |
| --- | --- | --- | --- | --- | --- | --- |
| Within ART | | GROW-Adjusted  Birth Weight  (adjusted for gestation, sex, parity) | Year of Conception Treatment Clinic | Fresh or Frozen Embryo Transfer  Deprivation Quintile  Smoking  Maternal Age (quadratic)  Paternal Age (quadratic)  ICSI (Y/N)  Number of Previous ART Cycles  Infertility Causes (x5) | GROW ABW: Table 2  Other Outcomes: Supplementary 6 | |
|  |  | Gestation |  | As above plus  Parity (Y/N) |  |  |
|  |  | Sex Ratio |  |  |  |  |
|  |  | NICU Admission |  |  |  |  |
|  |  | NICU Admission | Year of Conception Treatment Clinic  Gestation  Birth Weight |  | Not presented  as table | |
|  |  | GROW-Adjusted  Birth Weight  (adjusted for gestation, sex, parity) |  | As above plus:  *Culture Time*  *N^o^ of Embryos Created* | Not presented  as table | |
|  |  | 6-8 Weeks  > Weight | Age at Measurement  Treatment Clinic  Gender  Parity | Fresh or Frozen Embryo Transfer  Deprivation Quintile  Smoking  Maternal Age (quadratic)  Paternal Age (quadratic)  ICSI (Y/N)  Number of Previous ART Cycles  Infertility Causes (x5) | Supplementary 9 | |
|  |  | School Entry  > Weight  > Height  > BMI |  |  |  |  |
|  |  | 6-8 Weeks  > Weight |  | As above plus:  *Culture Time*  *N^o^ of Embryos Created* | Not presented as table | |
|  |  | School Entry  > Weight  > Height  > BMI |  |  |  |  |
|  |  | Growth Rate  > Birth - 6/8 wks  > 6/8 wks - PSE | Treatment Clinic  Gender  Parity | Deprivation Quintile  Smoking  Maternal Age (quadratic)  Paternal Age (quadratic)  ICSI (Y/N)  Number of Previous ART Cycles  Infertility Causes (x5) | Supplementary  10 | |
| ART vs. Naturally-Conceived | | GROW-Adjusted  Birth Weight  (adjusted for gestation, sex) | *Birth Year and Month; Gender;*  *Delivery Hospital*  *(Matching Variables)* | Naturally Conceived/ ART Fresh/ FET  Deprivation Quintile  Smoking  Maternal Age (quadratic) | GROW ABW: Table 1  Other Outcomes: Supplementary 5 | |
|  |  | Gestation |  |  |  |  |
|  |  | Sex Ratio |  |  |  |  |
|  |  | NICU Admission |  |  |  |  |
|  |  | GROW-Adjusted  Birth Weight  (adjusted for gestation, sex) | *Gender*  *Delivery Hospital* | Deprivation Quintile  Smoking  Maternal Age (quadratic)  *Year of delivery* | Not presented as table | |
|  |  | 6-8 Weeks  > Weight | *Birth Year and Month; Gender;*  *Delivery Hospital*  *(Matching variables)*  Age at measurement | Naturally Conceived/ ART Fresh/ FET  Deprivation Quintile  Smoking  Maternal Age (quadratic)  Feed Type @ 10 Days | Table 1  Supplementary 7/ 8 | |
|  |  | School Entry  > Weight  > Height  > BMI |  |  |  |  |
|  |  | Growth Rate  > Birth - 6/8 wks  > 6/8 wks - PSE |  | Naturally Conceived/ ART Fresh/ FET  Deprivation Quintile  Smoking  Maternal Age (quadratic)  Gender  Feed Type @ 10 Days | 3 | |
|  | | | | |  |  |

**Table S5:** Birth Outcomes for Fresh ART, Frozen ART (FET) and Naturally-Conceived Singleton Babies.

|  | **Raw Birth Weight**  N = 26,025 | | **Occipital Frontal Circumference**  N=21,169 | | **Crown-Heel Length**  N=15,427 | |
| --- | --- | --- | --- | --- | --- | --- |
|  | Effect size (95%CI) (g) | P | Effect size (95%CI) (mm) | P | Effect size (95%CI) (cm) | P |
| Type of Conception  Naturally Conceived  ART - Fresh Cycle  ART - Frozen Cycle | Reference  -143.5 (-164.5, -123.8)  18.2 (-16.6, 54.9) | <0.001 | Reference  -2.87 (-3.48, -2.24)  1.09 (0.06, 2.21) | <0.001 | Reference  -0.36 (-0.50, -0.22)  0.21 (-0.05, 0.47) | <0.001 |
| Scottish Index of Multiple Deprivation Quintile  1 (most deprived)  2  3  4  5 (least deprived) | -87.0 (-111.1, -65.0)  -53.8 (-77.3, -32.0)  -34.7 (-58.0, -12.3)  -5.7 (-28.0, 14.2)  Reference | <0.001 | -1.17 (-1.84, -0.49)  -0.88 (-1.56, -0.24)  -0.25 (-0.86, 0.35)  -0.04 (-0.58, 0.58)  Reference | 0.002 | 0.13 (-0.04, 0.30)  0.16 (-0.01, 0.31)  0.10 (-0.04, 0.24)  -0.01 (-0.14, 0.13)  Reference | 0.189 |
| Smoked during Pregnancy | -259.0 (-278.6, -240.4) | <0.001 | -4.61 (-5.23, -4.09) | <0.001 | -1.05 (-1.18, -0.92) | <0.001 |
| Maternal Age (Linear)  Maternal Age (Quadratic) | 18.6 (4.2, 31.8)  -34.4 (-51.7, -17.8) | <0.001 | 0.99 (0.60, 1.34)  -0.31 (-0.80, 0.19) | <0.001 | 0.20 (0.10, 0.30)  -0.14 (-0.26, -0.02) | <0.001 |

|  | **Sex Ratio**  N=26,034 | | **Unadjusted NICU Admission**  N = 25,608 | | **Gestation**  N=26,019 | |
| --- | --- | --- | --- | --- | --- | --- |
|  | Odds Ratio (95%CI) | P | Odds Ratio (95%CI) | P | Effect size (95%CI) (d) | P |
| Type of Conception  Naturally Conceived  ART - Fresh Cycle  ART - Frozen Cycle | Reference  1.007 (0.936, 1.083)  1.000 (0.880, 1.135) | 0.983 | Reference  1.24 (1.10, 1.40)  1.00 (0.80, 1.26) | 0.002 | Reference  -0.28 (-0.36, -0.20)  -0.17 (-0.31, -0.04) | <0.001 |
| Scottish Index of Multiple Deprivation Quintile  1 (most deprived)  2  3  4  5 (least deprived) | 0.997 (0.919, 1.082)  1.025 (0.944, 1.113)  0.956 (0.882, 1.036)  0.996 (0.921, 1.078)  Reference | 0.574 | 1.18 (1.03, 1.36)  1.05 (0.91, 1.22)  1.08 (0.93, 1.24)  0.90 (0.78, 1.04)  Reference | 0.006 | -0.22 (-0.30, -0.15)  -0.14 (-0.22, -0.07)  -0.14 (-0.21, -0.07)  -0.04 (-0.11, 0.03)  Reference | <0.001 |
| Smoked during Pregnancy | 1.073 (1.004, 1.147) | 0.038 | 1.37 (1.23, 1.53) | <0.001 | -0.32 (-0.39, -0.26) | <0.001 |
| Maternal Age (Linear)  Maternal Age (Quadratic) | 1.003 (0.956, 1.052)  1.016 (0.958, 1.077) | 0.874 | 1.09 (1.00, 1.18)  1.12 (1.02, 1.24) | 0.021 | -0.12 (-0.17, -0.07)  -0.08 (-0.14, -0.02) | <0.001 |

Results from Multiple Linear or Logistic Regression Models for Raw Birth Weight, Occipital Frontal Circumference and Crown-Heel Length, Sex Ratio, Neonatal Intensive Care Unit (NICU) Admission and Gestation at Birth, adjusting for Type of Conception, Scottish Index of Multiple Deprivation, Maternal Age and Smoking Status during Pregnancy and the ART-NC Matching Variables: Gender, Year and Month of Delivery and Delivery Hospital.

**Table S6**: Birth Outcomes in Fresh and Frozen Cycle ART-Conceived Singleton Babies: Association with ART treatment parameters.

|  | **Raw Birth Weight**  N=5,071 | | **Occipital Frontal Circumference**  N=4,104 | | **Crown Heel Length**  N=2,979 | |
| --- | --- | --- | --- | --- | --- | --- |
|  | Effect size (95%CI) (g) | P | Effect size (95%CI) (cm) | P | Effect size (95%CI) (cm) | P |
| Frozen Embryo-Transfer Cycle | 138.6 (94.0, 190.9) | <0.001 | 3.60 (2.50, 4.69) | 0.094 | 0.477 (0.194, 0.772) | <0.001 |
| Scottish Index of Multiple Deprivation Quintile  1 (most deprived)  2  3  4  5 (least deprived) | -117.3 (-178.0, -57.4)  -42.2 (-97.9, 13.3)  -41.6 (-91.7, 8.8)  -17.7 (-60.2. 26.4)  Reference | 0.005 | -0.63 (-2.21, 0.79)  -0.25 (-1.56, 1.07)  -0.25 (-1.41, 0.96)  -0.17 (-1.31, 0.82)  Reference | 0.121 | -0.013 (-0.431, 0.434)  0.057 (-0.305, 0.414)  0.097 (-0.205, 0.379)  -0.069 (-0.329, 0.190)  Reference | 0.218 |
| Smoked during Pregnancy | -211.9 (-274.8, -145.3) | <0.001 | -3.02 (-4.52, -1.39) | 0.024 | -0.667 (-1.062, -0.301) | <0.001 |
| Maternal Age (Linear)  Maternal Age (Quadratic) | -31.7 (-83.2, 19.3)  21.0 (-52.9, 93.4) | 0.400 | 0.47 (-0.77, 1.73)  -0.86 (-2.66, 0.90) | 0.033 | 0.171 (-0.145, 0.507)  0.301 (-0.194, 0.828) | 0.234 |
| Paternal Age (Linear)  Paternal Age (Quadratic) | 24.0 (-14.2, 60.1)  -11.8 (-41.7, 17.8) | 0.547 | 0.33 (-0.52, 1.29)  -0.08 (-0.89, 0.65) | 0.144 | -0.061 (-0.294, 0.176)  0.070 (-0.130, 0.258) | 0.681 |
| Intra-Cytoplasmic Sperm Injection (Y/ N) | 3.8 (-43.1, 50.5) | 0.878 | 0.12 (-1.13, 1.31) | 0.528 | 0.087 (-0.198, 0.387) | 0.674 |
| N^o^. Previous ART Cycles  0  1  2  3 or more | Reference  5.7 (-38.8, 49.7)  -13.6 (-70.1, 37.1)  -31.6 (-86.5, 16.4) | 0.528 | Reference  0.24 (-0.79,1.28)  -0.18 (-1.45, 1.12)  0.97 (-0.32, 2.18) | 0.063 | Reference  0.022 (-0.264, 0.300)  -0.006 (-0.298, 0.304)  -0.004 (-0.342, 0.319) | 0.875 |
| Infertility Cause  Fallopian Tubal Damage  Endometrial  Ovulatory Disorders  Male-Factor Cause  Idiopathic | -40.2 (-96.2, 17.8)  -43.2 (-109.6, 13.4)  -29.8 (-93.9, 33.5)  15.9 (-32.6, 67.0)  3.9 (-54.3, 61.0) | 0.177  0.170  0.359  0.532  0.895 | -0.33 (-1.81, 1.06)  0.36 (-1.50, 2.19)  -0.11 (-1.77, 1.56)  0.17 (-1.15, 1.46)  -0.40 (-1.85, 0.96) | 0.167  0.382  0.607  0.155  0.038 | 0.142 (-0.216, 0.502)  -0.192 (-0.635, 0.265)  0.043 (-0.383, 0.443)  0.203 (-0.148, 0.534)  0.137 (-0.232, 0.520) | 0.555  0.482  0.426  0.527  0.070 |

**Table S6 Continued**:

|  | **Sex Ratio**  N=5,071 | | **Unadjusted NICU Admission**  N = 4,997 | | **Gestation**  N=5,071 | |
| --- | --- | --- | --- | --- | --- | --- |
|  | Odds Ratio (95%CI) | P | Odds Ratio (95%CI) | P | Effect size (95%CI) (d) | P |
| Frozen Embryo-Transfer Cycle | 0.987 (0.843, 1.156) | 0.873 | 0.89 (0.68, 1.18) | 0.422 | 0.138 (-0.022, 0.301) | 0.094 |
| Scottish Index of Multiple Deprivation Quintile  1 (most deprived)  2  3  4  5 (least deprived) | 0.946 (0.769, 1.163)  1.104 (0.919, 1.326)  0.929 (0.788, 1.094)  1.027 (0.884, 1.192)  Reference | 0.473 | 0.99 (0.70, 1.39)  0.87 (0.63, 1.19)  1.09 (0.84, 1.43)  0.91 (0.71, 1.18)  Reference | 0.672 | -0.279 (-0.516, -0.046)  -0.063 (-0.272, 0.128)  -0.139 (-0.310, 0.044)  -0.012 (-0.150, 0.136)  Reference | 0.121 |
| Smoked during Pregnancy | 1.060 (0.865, 1.298) | 0.574 | 1.15 (0.83, 1.59) | 0.397 | -0.256 (-0.469, -0.045) | 0.024 |
| Maternal Age (Linear)  Maternal Age (Quadratic) | 1.221 (1.023, 1.458)  1.086 (0.822, 1.434) | 0.084 | 1.44 (1.01, 2.04)  0.72 (0.41, 1.28) | 0.029 | -0.219 (-0.405, -0.029)  -0.170 (-0.409, 0.100) | 0.033 |
| Paternal Age (Linear)  Paternal Age (Quadratic) | 1.001 (0.878, 1.141)  0.953 (0.847, 1.073) | 0.678 | 0.82 (0.64, 1.06)  1.04 (0.82, 1.31) | 0.161 | 0.113 (-0.019, 0.249)  0.003 (-0.112, 0.112) | 0.144 |
| Intra-Cytoplasmic Sperm Injection (Y/ N) | 1.129 (0.952, 1.339) | 0.164 | 1.10 (0.82, 1.47) | 0.525 | 0.058 (-0.129, 0.232) | 0.528 |
| N^o^. Previous ART Cycles  0  1  2  3 or more | Reference  0.951 (0.819, 1.106)  1.195 (0.998, 1.432)  1.101 (0.920, 1.318) | 0.077 | Reference  0.90 (0.70, 1.16)  0.95 (0.70, 1.29)  0.91 (0.68, 1.24) | 0.873 | Reference  0.015 (-0.141, 0.177)  0.025 (-0.173, 0.218)  -0.215 (-0.414, -0.012) | 0.063 |
| Infertility Cause  Fallopian Tubal Damage  Endometrial  Ovulatory Disorders  Male-Factor Cause  Idiopathic | 1.235 (1.011, 1.508)  1.062 (0.843, 1.338)  1.138 (0.900, 1.439)  1.056 (0.881, 1.267)  1.134 (0.925, 1.391) | 0.038  0.611  0.281  0.554  0.225 | 1.23 (0.89, 1.70)  0.92 (0.63, 1.36)  1.18 (0.81, 1.72)  0.76 (0.57, 1.02)  0.81 (0.58, 1.14) | 0.218  0.690  0.395  0.068  0.229 | -0.149 (-0.360, 0.068)  -0.107 (-0.349, 0.112)  -0.061 (-0.298, 0.167)  0.135 (-0.043, 0.315)  0.220 ( 0.011, 0.422) | 0.167  0.382  0.607  0.155  0.038 |

Results from Multiple Linear or Logistic Regression Models for Raw Birth Weight, Occipital Frontal Circumference and Crown-Heel Length, Sex Ratio, Neonatal Intensive Care Unit (NICU) Admission and Gestation, adjusting for Type of ART Conception, Scottish Index of Multiple Deprivation, Maternal Age and Smoking Status during Pregnancy, Paternal Age, whether or not ICSI was used, the Number of Previous ART Cycles and Causes of Infertility.

**Table S7:** Infant weight, occipital frontal circumference (OFC) and length at 6-8 weeks in ART and Naturally-Conceived Singleton Babies.

|  | **6-8 weeks** | | | | | |
| --- | --- | --- | --- | --- | --- | --- |
|  | **Weight**  N=19,028 | | **OFC**  N=18,873 | | **Length**  N=18,870 | |
|  | Effect size (95%CI) (g) | P | Effect size  (95% CI) (mm) | P | Effect size  (95% CI) (cm) | P |
| Type of Conception  Naturally Conceived  ART - Fresh Cycle  ART - Frozen Cycle | Reference  -171 (-200, -143)  1 (-47, 49) | 0.340 | Reference  -2.41 (-3.05, -1.87)  -0.41 (-1.51, 0.63) | <0.001 | Reference  -0.39 (-0.52, -0.27)  0.08 (-0.15, 0.29) | <0.001 |
| Scottish Index of Multiple Deprivation Quintile  1 (most deprived)  2  3  4  5 (least deprived) | -48 (-80, -17)  -25 (-57, 4)  -32 (-61, 0)  -15 (-48, 13)  Reference | 0.131 | -2.00 (-2.68, -1.34)  -0.92 (-1.63, -0.22)  -0.61 (-1.30, 0.10)  -0.39 (-1.04, 0.27)  Reference | <0.001 | -0.19 (-0.33, -0.05)  -0.09 (-0.23, 0.06)  -0.09 (-0.23, 0.04)  -0.09 (-0.23, 0.04)  Reference | 0.115 |
| Smoked during Pregnancy | -190 (-216, -163) | 0.638 | -2.91 (-3.51, -2.33) | <0.001 | -1.00 (-1.12, -0.88) | <0.001 |
| Maternal Age (Linear)  Maternal Age (Quadratic) | -7 (-25, 12)  -52 (-75, -29) | 0.011 | 0.41 (-0.01, 0.79)  -0.51 (-1.03, -0.00) | 0.007 | -0.13 (-0.21, -0.05)  -0.12 (-0.22, -0.02) | 0.002 |
| Type of feeding  Breast only  Bottle only  Breast & Bottle | Reference  77 (54, 98)  -9 (-52, 30) | 0.125 | Reference  1.21 (0.74, 1.67)  -0.20 (-1.07, 0.69) | <0.001 | Reference  -0.00 (-0.10, 0.09)  -0.14 (-0.32, 0.03) | 0.300 |

Results from Multiple Linear Regression Models for Infant Weight, OFC and Length at 6-8 weeks, adjusting for Type of Conception, Scottish Index of Multiple Deprivation, Maternal Age and Smoking Status during Pregnancy, Feed Type at 10 Days and the ART-NC Matching Variables: Gender, Year and Month of Delivery and Delivery Hospital. Models also make adjustments for age-at-measurement.

**Table S8:** Weight, Height and Body Mass Index (BMI) at Primary School entry in ART and Naturally-Conceived Singleton Babies.

|  | **School Entry** | | | | | |
| --- | --- | --- | --- | --- | --- | --- |
|  | **Weight**  N=11,573 | | **Height**  N=11,545 | | **BMI**  N=11,560 | |
|  | Effect size  (95% CI) (g) | P | Effect size  (95% CI) (cm) | P | Effect size  (95% CI) (kg/m^2^) | P |
| Type of Conception  Naturally Conceived  ART - Fresh Cycle  ART - Frozen Cycle | Reference  -58 (-231, 115)  200 (-135, 560) | 0.340 | Reference  0.28 (-0.01, 0.53)  0.68 (0.25, 1.13) | 0.003 | Reference  -0.12 (-0.21, -0.03)  -0.04 (-0.22, 0.14) | 0.035 |
| Scottish Index of Multiple Deprivation Quintile  1 (most deprived)  2  3  4  5 (least deprived) | 38 (-168, 240)  229 (42, 430)  104 (-74, 294)  29 (-150, 201)  Reference | 0.131 | -0.85 (-1.15, -0.56)  -0.33 (-0.59, -0.05)  -0.17 (-0.44, 0.12)  -0.29 (-0.55, 0.00)  Reference | <0.001 | 0.25 (0.15, 0.37)  0.26 (0.16, 0.37)  0.13 (0.03, 0.24)  0.11 (0.01, 0.20)  Reference | <0.001 |
| Smoked during Pregnancy | -40 (-194, 136) | 0.638 | -0.85 (-1.08, -0.60) | <0.001 | 0.17 (0.09, 0.27) | <0.001 |
| Maternal Age (Linear)  Maternal Age (Quadratic) | 118 (3, 240)  -121 (-266, 17) | 0.011 | 0.21 (0.03, 0.38)  -0.13 (-0.34, 0.08) | 0.013 | 0.02 (-0.04, 0.08)  -0.05 (-0.13, 0.02) | 0.243 |
| Type of feeding  Breast only  Bottle only  Breast & Bottle | Reference  99 (-31, 229)  229 (-25, 501 | 0.125 | Reference  -0.20 (-0.39, 0.00)  0.29 (-0.10, 0.67) | 0.019 | Reference  0.12 (0.06, 0.19)  0.08 (-0.05, 0.22) | 0.002 |

Results from Multiple Linear Regression Models for Weight, Height and BMI at Primary School Entry, adjusting for Type of Conception, Scottish Index of Multiple Deprivation, Maternal Age and Smoking Status during Pregnancy, Feed Type at 10 Days and the ART-NC Matching Variables: Gender, Year and Month of Delivery and Delivery Hospital. Models also make adjustments for age-at-measurement.

**Table S9**: Infant Weight at 6-8 weeks and Weight, Height and Body Mass Index (BMI) at Primary School entry in Fresh and Frozen Cycle ART-Conceived Singleton Babies: Association with ART treatment parameters.

|  | **6-8 Weeks** | | **School Entry** | | | | | |
| --- | --- | --- | --- | --- | --- | --- | --- | --- |
|  | **Weight**  N=3,908 | | **Weight**  N=2,571 | | **Height**  N=2,564 | | **BMI**  N = 2,570 | |
|  | Effect size (95%CI) (g) | P | Effect size (95%CI) (g) | P | Effect size (95%CI) (cm) | P | Effect Size (95%CI) (kg/m^2^) | P |
| Frozen Embryo-Transfer Cycle | 133.3 (75.4, 190.2) | <0.001 | 257.8 (-115.5, 650.7) | 0.172 | 0.56 (0.07, 1.11) | 0.030 | 0.043 (-0.150, 0.233) | 0.665 |
| Scottish Index of Multiple Deprivation Quintile  1 (most deprived)  2  3  4  5 (least deprived) | 23.9 (-53.4, 101.9)  53.0 (-12.8, 118.3)  29.8 (-29.8, 92.7)  -16.4 (-74.6, 40.0)  Reference | 0.288 | 494.1 (-63.4, 1,051)  67.7 (-328.9, 464.3)  69.3 (-295.1, 431.7)  22.2 (-321.9, 323.1)  Reference | 0.502 | -0.26 (-0.95, 0.40)  -0.48 (-1.08, 0.08)  -0.24 (-0.78, 0.29)  -0.43 (-0.95, 0.05)  Reference | 0.410 | 0.406 ( 0.137, 0.702)  0.215 (-0.009, 0.430)  0.146 (-0.047, 0.351)  0.163 (-0.008, 0.337)  Reference | 0.042 |
| Smoked during Pregnancy | -117.9 (-197.4, -39.5) | 0.004 | 118.6 (-354.0, 667.8) | 0.649 | -1.08 (-1.79, -0.41) | 0.002 | 0.404 (0.100, 0.697) | 0.006 |
| Maternal Age (Linear)  Maternal Age (Quadratic) | -69.9 (-172.4, 41.2)  14.0 (-90.6, 110.7) | 0.225 | 205.9 (-440.8, 840.6)  -157.0 (-800.4, 448.9) | 0.818 | -0.21 (-1.28, 0.80)  0.40 (-0.57, 1.47) | 0.668 | 0.145 (-0.229, 0.464)  -0.167 (-0.474, 0.185) | 0.609 |
| Paternal Age (Linear)  Paternal Age (Quadratic) | -32.6 (-80.1, 17.0)  11.2 (-35.3, 58.0) | 0.423 | -19.0 (-306.9, 280.7)  122.9 (-192.7, 504.1) | 0.750 | 0.12 (-0.32, 0.56)  -0.07 (-0.54, 0.40) | 0.866 | -0.017 (-0.181, 0.134)  0.099 (-0.061, 0.263) | 0.455 |
| Intra-Cytoplasmic Sperm Injection (Y/ N) | 27.4 (-27.3, 84.3) | 0.347 | 124.8 (-270.9, 551.2) | 0.543 | 0.03 (-0.52, 0.57) | 0.916 | 0.090 (-0.129, 0.294) | 0.402 |
| N^o^. Previous ART Cycles  0  1  2  3 or more | Reference  23.6 (-35.6, 77.6)  -7.4 (-76.1, 61.3)  -5.7 (-75.7, 61.2) | 0.764 | Reference  195.3 (-149.9, 537.8)  -18.4 (-427.5, 390.0)  -44.9 (-461.8, 342.2) | 0.598 | Reference  -0.20 (-0.65, 0.26)  0.00 (-0.58, 0.54)  -0.17 (-0.75, 0.40) | 0.823 | Reference  0.179 ( 0.002, 0.368)  -0.042 (-0.236, 0.163)  0.007 (-0.201, 0.224) | 0.158 |
| Infertility Cause  Fallopian Tubal Damage  Endometrial  Ovulatory Disorders  Male-Factor Cause  Idiopathic | -34.2 (-112.6, 40.1)  -71.0 (-148.0, 13.1)  -37.8 (-125.7, 50.2)  -28.1 (-90.4, 32.4)  -44.2 (-114.6, 35.7) | 0.370  0.074  0.409  0.364  0.229 | -210.3 (-610.3, 229.3)  -470.8 (-915.2, -33.0)  -88.8 (-604.9, 408.4)  -12.1 (-375.0, 352.7)  -272.0 (-673.0, 173.5) | 0.344  0.042  0.725  0.949  0.206 | -0.23 (-0.87, 0.40)  -0.46 (-1.14, 0.19)  -0.26 (-1.04, 0.48)  0.08 (-0.48, 0.60)  -0.24 (-0.83, 0.41) | 0.476  0.184  0.508  0.786  0.452 | -0.083 (-0.302, 0.159)  -0.193 (-0.452, 0.039)  0.012 (-0.266, 0.299)  -0.040 (-0.238, 0.164)  -0.129 (-0.361, 0.121) | 0.475  0.130  0.934  0.701  0.287 |

Results from Multiple Linear Regression Models for Weight at 6-8 weeks and Weight, Height and BMI at Primary School Entry, adjusting for Type of ART Conception, Scottish Index of Multiple Deprivation, Maternal Age and Smoking Status during Pregnancy, Paternal Age, whether or not ICSI was used, the Number of Previous ART Cycles and Causes of Infertility. All models are also adjusted for age at measurement and Infertility Treatment Centre.

**Table S10**: Growth Rates of Singleton Births from Fresh and Frozen (FET) ART Transfers between Birth and 6-8 Weeks and 6-8 Weeks and Primary School Entry: Association with ART treatment parameters.

|  | **Birth/ 6-8 Weeks** | | **6-8 Weeks/ School Entry** | |
| --- | --- | --- | --- | --- |
|  | Coefficient (95% C.I.) | p | Coefficient (95% C.I.) | p |
| Frozen Embryo-Transfer Cycle | -7.4 (-19.0, 3.8) | 0.218 | 1.03 (-0.33, 2.33) | 0.141 |
| Scottish Index of Multiple Deprivation Quintile  1 (most deprived)  2  3  4  5 (least deprived) | 22.8 (8.1, 39.0)  10.5 (-2.9, 23.6)  6.5 (-6.1, 18.7)  4.8 (-6.2, 15.5)  Reference | 0.059 | 1.21 (-0.56, 3.13)  0.20 (-1.20, 1.60)  -0.26 (-1.53, 0.98)  0.32 (-0.84, 1.62)  Reference | 0.579 |
| Smoked during Pregnancy | 24.1 (7.2, 41.1) | 0.007 | 0.13 (-1.86, 1.98) | 0.890 |
| Feed Type at 10 Days  Breast  Bottle  Breast & Bottle | Reference  27.9 (19.5, 37.6)  6.2 (-7.2, 19.7) | <0.001 | Reference  -0.11 (-1.13, 0.84)  0.67 (-0.72, 2.06) | 0.588 |
| Maternal Age (Linear)  Maternal Age (Quadratic) | -8.3 (-31.4, 12.6)  5.0 (-14.9, 27.9) | 0.737 | 0.57 (-1.97, 2.83)  0.01 (-2.33, 2.37) | 0.711 |
| Paternal Age (Linear)  Paternal Age (Quadratic) | -7.4 (-18.3, 1.9)  -0.2 (-8.9, 8.5) | 0.248 | -0.25 (-1.28, 0.93)  0.65 (-0.49, 1.79) | 0.537 |
| Intra-Cytoplasmic Sperm Injection (Y/ N) | 0.5 (-12.1, 13.4) | 0.946 | 0.62 (-0.81, 2.01) | 0.401 |
| N^o^. Previous ART Cycles  0  1  2  3 or more | Reference  1.0 (-10.3, 12.3)  3.8 (-10.1, 16.8)  11.5 (-3.7, 26.2) | 0.415 | Reference  0.44 (-0.75, 1.63)  -0.24 (-1.62, 1.25)  -0.66 (-2.16, 0.78) | 0.523 |
| Infertility Cause  Fallopian Tubal Damage  Endometrial  Ovulatory Disorders  Male-Factor Cause  Idiopathic | 11.9 (-3.8, 25.5)  4.4 (-13.6, 22.4)  9.9 (-8.0, 27.6)  -3.4 (-15.0, 8.1)  -7.5 (-22.2, 6.9) | 0.102  0.625  0.268  0.562  0.312 | -0.16 (-1.62, 1.40)  -1.25 (-2.74, 0.32)  -0.60 (-2.41, 1.29)  0.19 (-1.18, 1.48)  -0.36 (-1.80, 1.20) | 0.834  0.110  0.521  0.782  0.643 |

Results from Multiple Linear Regression Models for Growth (Change in Weight) between Birth and 6-8 Weeks (average weekly growth rate) and 6-8 Weeks and Primary School Entry (average monthly growth rate), adjusting for Type of ART Conception, Scottish Index of Multiple Deprivation, Maternal Age and Smoking Status during Pregnancy, Feed Type at 10 Days, Paternal Age, whether or not ICSI was used, the Number of Previous ART Cycles and Causes of Infertility.
